# Endothelial microparticles prevent lipid-induced endothelial damage *via* Akt/eNOS signaling and reduced oxidative stress

**DOI:** 10.1096/fj.201601244RR

**Published:** 2017-07-07

**Authors:** Ayman M. Mahmoud, Fiona L. Wilkinson, Eoghan M. McCarthy, Daniel Moreno-Martinez, Alexander Langford-Smith, Miguel Romero, Juan Duarte, M. Yvonne Alexander

**Affiliations:** * Healthcare Science Research Centre, Faculty of Science and Engineering, Manchester Metropolitan University, Manchester, United Kingdom;; † Manchester Academic Health Science Centre, Manchester, United Kingdom;; ‡ Physiology Division, Department of Zoology, Faculty of Science, Beni-Suef University, Beni Suef, Egypt;; § Centre for Musculoskeletal Research, Institute of Inflammation and Repair, Manchester Academic Health Science Centre, University of Manchester, Manchester, United Kingdom;; ¶ Musculoskeletal Biomedical Research Unit, National Institute for Health Research Manchester, Central Manchester University Hospital NHS Foundation Trust, Manchester, United Kingdom;; ║ Department of Pharmacology, School of Pharmacy, University of Granada, Granada, Spain;; # Instituto de Investigación Biosanitaria de Granada, Granada, Spain

**Keywords:** extracellular microvesicles, endothelial dysfunction, Nrf2

## Abstract

Endothelial microparticles (EMPs) are endothelium-derived submicron vesicles that are released in response to diverse stimuli and are elevated in cardiovascular disease, which is correlated with risk factors. This study investigates the effect of EMPs on endothelial cell function and dysfunction in a model of free fatty acid (FFA) palmitate-induced oxidative stress. EMPs were generated from TNF-α-stimulated HUVECs and quantified by using flow cytometry. HUVECs were treated with and without palmitate in the presence or absence of EMPs. EMPs were found to carry functional eNOS and to protect against oxidative stress by positively regulating eNOS/Akt signaling, which restored NO production, increased superoxide dismutase and catalase, and suppressed NADPH oxidase and reactive oxygen species (ROS) production, with the involvement of NF-erythroid 2-related factor 2 and heme oxygenase-1. Conversely, under normal conditions, EMPs reduced NO release and increased ROS and redox-sensitive marker expression. In addition, functional assays using EMP-treated mouse aortic rings that were performed under homeostatic conditions demonstrated a decline in endothelium-dependent vasodilatation, but restored the functional response under lipid-induced oxidative stress. These data indicate that EMPs harbor functional eNOS and potentially play a role in the feedback loop of damage and repair during homeostasis, but are also effective in protecting against FFA-induced oxidative stress; thus, EMP function is reflected by the microenvironment.—Mahmoud, A. M., Wilkinson, F. L., McCarthy, E. M., Moreno-Martinez, D., Langford-Smith, A., Romero, M., Duarte, J., Alexander, M. Y. Endothelial microparticles prevent lipid-induced endothelial damage *via* Akt/eNOS signaling and reduced oxidative stress.

Endothelial dysfunction underpins the progression of cardiovascular disease (1, 2) and is present in patients with type 2 diabetes (3). Classic hallmarks of endothelial dysfunction are reduced NO bioavailability, impaired endothelial-mediated vasorelaxation, inflammation, elevated reactive oxygen species (ROS), hemodynamic deregulation, and high circulating levels of free fatty acids (FFAs) (4–7). Palmitate—the main saturated FFA in the bloodstream—induces ROS production in the vasculature *via* increased levels and activity of NADPH oxidases, mitochondrial uncoupling (8, 9) and down-regulation of eNOS (10).

NF-erythroid 2–related factor 2 (Nrf2), a redox-sensitive transcription factor, is crucial for the regulation of the expression of various antioxidant genes *via* binding to the antioxidant response element (ARE) that is present in NAD(P)H:quinone oxido-reductase 1 (NQO1), heme oxygenase-1 (HO-1), glutathione peroxidase, and the superoxide dismutase (SOD) family (11–13).

The term “extracellular vesicles” has been used to include 3 categories of extracellular microparticles (MPs); apoptotic bodies, microvesicles/MPs, and exosomes, depending on their size. Microvesicles are gaining interest as cell-derived sealed membrane vesicles that act as vehicles for the intercellular transfer of mRNAs, microRNAs, lipids, and proteins, and reflecting their cell of origin. This allows cell communication either in a paracrine or endocrine fashion. MPs are small-membrane vesicles (100–1000 nm) that are released from different cell types under adverse conditions, including turbulent flow and proapoptotic stimulation (14). MPs can also be released in small numbers under basal physiologic conditions (15). Our laboratory has focused, in particular, on 100–1000 nm microvesicles that are released from endothelial cells, which we describe as endothelial microparticles (EMPs) (16–20). Although eNOS has been identified on EMPs (21), there is no evidence that supports functional eNOS activity (22), which we now show for the first time. MPs have been reported to be taken up by either membrane fusion, receptor-mediated cell signaling, or phagocytosis (23, 24) and are now recognized as important circulating biologic vectors with potential as surrogate markers for multiple pathophysiologic conditions (25). Indeed, EMPs have been associated with several cardiovascular risk factors (26), as well as increased production of ROS in rat aortic rings (27) and endothelial cells *in vitro* (28, 29). Despite links with impaired endothelial function (28) and apoptosis (30), EMPs have also been reported to protect endothelial cells against damage by promoting cell survival *via* induction of cytoprotective and anti-inflammatory effects (17, 18, 31).

We hypothesize that EMPs have a dual function depending on the physiologic conditions. We used an *in vitro* and *ex vivo* endothelial cell model of lipid-induced oxidative stress by treating HUVECs and mouse aortic rings with palmitate and investigated several parameters after exposure to EMPs in terms of endothelial signaling and function, which may be triggered in the process.

## MATERIALS AND METHODS

### HUVEC culture

HUVECs from pooled donors (Caltag Medsystems, Buckingham, United Kingdom) were cultured in Medium 199 (Lonza, Brussels, Belgium) that was supplemented with 20% fetal bovine serum, penicillin/streptomycin (2 mM), glutamine (2 mM), HEPES (10 mM), endothelial cell growth supplement (30 μg/ml), and heparin (100 µg/ml) under 5% CO_2_ at 37°C. Experiments were repeated in 3 different populations of HUVECs from passage 2 to 7, with no differences observed between passages or populations.

### Generation and isolation of EMPs

Confluent HUVECs were incubated in complete medium and treated with 10 ng/ml TNF-α (PromoCell, Heidelberg, Germany) for 24 h under 5% CO_2_ at 37°C. Conditioned medium was collected and cleared from detached cells and cell fragments by centrifugation at 4300 *g* for 5 min at room temperature. Supernatants were transferred to thick-wall polycarbonate ultracentrifugation tubes (Beckman Coulter, High Wycombe, United Kingdom) and centrifuged at 10^5^ *g* for 2 h at 4°C using an Optima XE ultracentrifuge (Beckman Coulter), as described previously (32). Pellets were carefully washed in PBS and centrifuged again under the same conditions. Washed pellets that contained MPs were resuspended in PBS. As such, the preparation may contain microvesicles/exosomes. We define EMPs in this manuscript specifically by the membrane particles from the cell of origin after this centrifugation step.

EMP quantification was carried out by using an established protocol in our laboratory (17). See the Supplemental Data for details.

### Confirmation of EMP purity

Simultaneous incubation for 15 min with fluorescent Abs was performed by using 2.5 µl of phycoerythrin-conjugated anti-human CD31 (55546; BD Pharmingen, San Diego, CA, USA), 2.5 µl of allophycocyanin-conjugated anti-human CD42b (551061; BD Pharmingen), and 5 µl of FITC-conjugated anti-human annexin-V marker (51-65874X; BD Pharmingen). Flow cytometry was performed on prepared samples by using a FACSVerse flow cytometer (BD Biosciences, San Jose, CA, USA). Analysis was stopped once 1000 beads had been counted, and gates were set to exclude artifacts and beads. Events that were positive for annexin-V (MP marker) and CD31 (endothelial marker) and negative for CD42b (platelet-marker; annexin- V^+^/CD31^+^/CD42b^−^) were defined as EMPs. The purity of this isolation is >98%, which indicates that the isolated MPs are of endothelial cell origin.

### Palmitate and EMP treatment

Lipid-containing media were prepared by conjugation of sodium palmitate to bovine serum albumin (BSA) according to Chavez and Summers (33), with some modifications. In brief, palmitate was dissolved in ethanol in a 60°C water bath before diluting 1:100 in M199 that contained 2% (wt/vol) fatty acid-free BSA and prewarmed for 1 h at 37°C. On the basis of our initial optimization studies (34) and our previous work where levels of 10^5^ and 10^6^ EMPs/ml were detected in patients with systemic lupus erythematosus (17) and carotid artery disease (19), respectively, these 2 doses were chosen for EMP treatment. A concentration of 10^5^ EMPs/ml was also used to assess for a concentration–dose response. Therefore, 5 × 10^3^ HUVECs/cm^2^ (confluent) were cultured in complete medium and treated with 10^5^ or 10^6^ EMPs/ml for 24 h or coincubated with palmitate (100 µM) for 24 h, or with palmitate added for the last 3 h of the incubation period. Control cells were incubated in serum-free M199 that contained 2% (wt/vol) fatty acid-free BSA.

### Assay of NO release by HUVECs and EMPs

NO released by HUVECs was quantified by using the NO-sensitive fluorescent probe, diaminofluorescein-2 (DAF-2), as described previously (35). After incubation, cells were washed with PBS and preincubated with l-arginine (100 μM in PBS) for 5 min at 37°C. In some experiments, L-NAME (*N*-nitroarginine methyl ester; 100 μM) was added 20 min before the addition of l-arginine. Cells were then incubated with DAF-2 (0.1 μM) for 2 min, followed by the calcium ionophore, calimycin (A23187; 1 μM), for 30 min. Fluorescence intensity (arbitrary units) was measured by using a microplate reader (BioTek Instruments, Winooski, VT, USA), and autofluorescence was subtracted from each value. The difference between fluorescence signal with and without L-NAME was considered NO production.

The same procedure was followed to assay the NO release from EMPs that were resuspended in PBS at 10^5^ and 10^6^/well of a 96-well plate.

### Assay of intracellular ROS production

Confluent HUVECs (96-well plates) were incubated with 10 µM of the fluorescent probe, CMH_2_DCF-DA (2′-7′-dichlorodihydrofluorescein diacetate; Sigma-Aldrich, St. Louis, MO, USA), for 30 min at 37°C. Fluorescence intensity was measured at excitation 490 nm and emission 540 nm by using a microplate reader (BioTek Instruments).

### Assay of NADPH oxidase activity

HUVECs and mouse aortic rings were incubated with 10^5^ or 10^6^ EMPs for 24 h in the presence or absence of palmitate. Palmitate treatment was performed as the model of chronic or acute lipid-induced oxidative stress either for 24 h in coculture with EMPs or simply as a final 3-h stimulation in EMP-pretreated HUVECs before harvesting at 24 h, respectively. NADPH-enhanced superoxide (O_2_^⋅−^) release in homogenates from cultured HUVECs or mouse aortic rings was quantified by lucigenin-enhanced chemiluminescence, as previously described (36).

### Measurement of lipid peroxidation

Malondialdehyde (MDA)—an index for determining the extent of lipid peroxidation—was measured by using OxiSelect thiobarbituric acid reactive species assay kit (Cell Biolabs, San Diego, CA, USA) according to manufacturer instructions. To prevent artifactual oxidation of lipids during sample processing and the thiobarbituric acid reaction, we added butylated hydroxytoluene to the thiobarbituric acid reagent and samples.

### Determination of SOD and catalase activity

SOD and catalase (CAT) activity was determined in HUVEC homogenate by using Cayman’s assay kits (Cayman Chemical, Ann Arbor, MI, USA).

### RNA isolation and RT-PCR analysis

Total RNA was extracted from HUVECs by using Trizol reagent (Thermo Fisher Scientific, Waltham, MA, USA). RNA samples were quantified at 260 nm. RNA samples with *A*_260_/*A*_280_ ratios ≥1.7 were selected. First-strand cDNAs were synthesized from 2 µg total RNA by using SuperScript II reverse transcriptase and oligo deoxythymidine primers (Sigma-Aldrich). Reverse transcription was performed with a Surecycler 8800 thermocycler (Agilent Technologies, Santa Clara, CA, USA), and reverse transcription products were amplified by SYBR Green master mix (BioLine, London, United Kingdom) in a total volume of 20 µl by using the primer set and conditions described previously (36) (Supplemental Data). Housekeeping gene GAPDH (glyceraldehyde 3-phosphate dehydrogenase) was used for normalizing gene expression.

### Western blot analysis

Total protein extracts were harvested from HUVECs and EMPs, lysed in RIPA buffer, supplemented with proteinase inhibitors, and analyzed by Western blotting as previously described (37) (Supplemental Data).

### *Ex vivo* assessment of mouse aortic ring vascular reactivity

Thoracic aortas were obtained from male BALB/c mice (Janvier, St. Berthevin Cedex, France). All procedures conformed to the Guide for the Care and Use of Laboratory Animals and were approved by the Institutional Committee for the Ethical Care of Animals (University of Granada, Granada, Spain). Wire myography experiments were performed as previously described (37) (Supplemental Data).

### Statistical analysis

Statistical analysis was performed by using Prism 5 (GraphPad Software, La Jolla, CA, USA) using 1-way ANOVA and Tukey’s *post hoc* analysis. A 2-factor ANOVA was used to test for group interactions. Results were expressed as means ± sem and *P* < 0.05 was considered significant. The number of independent experiments performed are stated in the figure legends.

## RESULTS

### EMPs carry a functional eNOS enzyme

**Figure 1.**
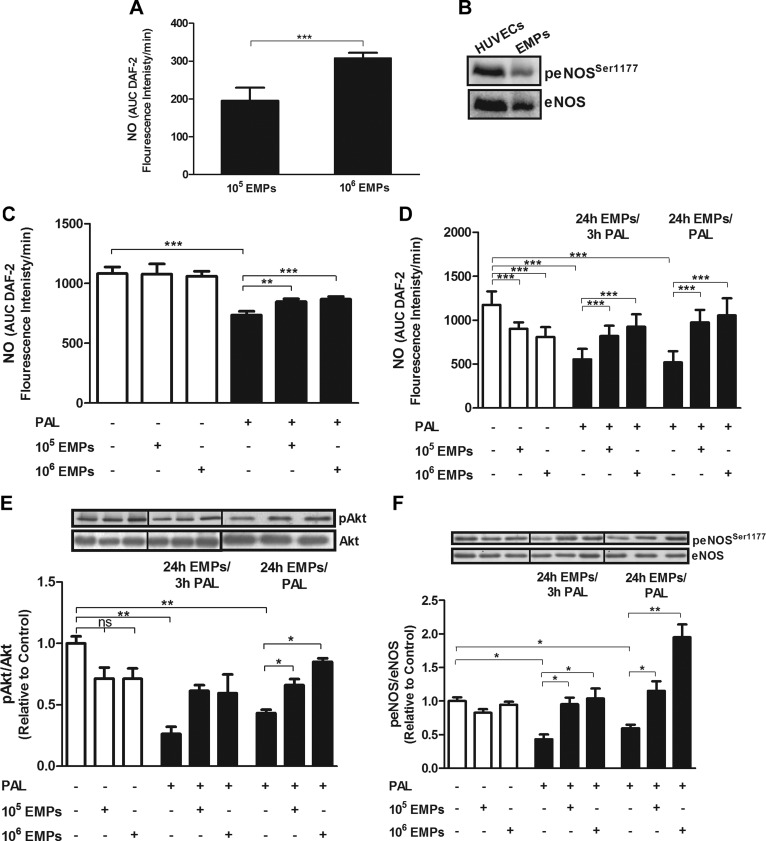
EMPs express a functional eNOS, elevate NO in palmitate (Pal)-induced HUVECs, and increase phosphorylation levels of Akt and eNOS. *A*) EMPs were incubated with l-arginine for 5 min at 37°C, followed by the addition of DAF-2, and NO production was determined. EMPs produce eNOS-derived NO in a concentration-dependent manner, which is inhibited by the NOS inhibitor, L-NAME. The difference between fluorescence signal with and without L-NAME is considered NO production. *B*) peNOS^Ser1177^ expression in EMPs and HUVECs as controls. *C*) HUVECs that were incubated with EMPs for 3 h showed no changes in NO production, whereas EMPs prevented the Pal-induced decline in NO production. *D*) Pal and EMPs diminish NO production. Treatment with either EMPs for 24 h with the addition of Pal during the last 3 h (24 h EMPs/3 h Pal) or with EMPs and Pal for 24 h (24 h EMPs/Pal) protects against Pal-induced reduction in NO production. Results are means ± sem; *n* = 8–12. *E*, *F*) Treatment of HUVECs with 100 µM Pal for either 3 or 24 h decreases protein phosphorylation of Akt (*E*) and eNOS at Ser1177 (*F*). EMPs up-regulate Akt and eNOS phosphorylation in HUVECs that are treated with either EMPs for 24 h with the addition of Pal during the last 3 h (24 h EMPs/3 h Pal) or with EMPs and Pal for 24 h (24 h EMPs/Pal). Results are means ± sem; *n* = 6 and analyzed using 1-way ANOVA. AUC, area under curve; ns, nonsignificant. *P < 0.05, **P < 0.01, ***P < 0.001.

To determine whether EMPs carry functional eNOS, 2 doses—10^5^ and 10^6^ EMPs—were incubated with the eNOS substrate, l-arginine, for 5 min at 37°C, followed by addition of DAF-2 to assay basal NO production. eNOS was then stimulated with the Ca^2+^ ionophore, A23187, and NO production was determined after 5, 15, and 30 min. EMPs caused an increase in eNOS-derived NO production in both doses, whereas addition of the NOS inhibitor, L-NAME, significantly impaired A23187-stimulated NO production (*P* < 0.001; **Fig. 1*A*** and Supplemental Fig. 1*A*).

The presence of peNOS in EMPs was further assessed by Western blot analysis using HUVECs as a control. peNOS^Ser1177^ and total eNOS were detected in the EMP lysate (Fig. 1*B*).

### EMPs have differential effects on NO production in HUVECs depending on the presence or absence of oxidative stress

We next established the effects of EMPs on HUVECs under normal conditions and FFA-induced oxidative stress by incubation with palmitate. To determine whether the duration of oxidative stress was important and whether EMPs exerted a protective or restorative effect under these conditions, NO production was measured in 5 treatment groups: *1*) HUVECs that were treated with EMPs alone for 3 h; *2*) HUVECs in the combined presence of EMPs and palmitate for 3 h; *3*) HUVECs that were treated with EMPs alone for 24 h; *4*) HUVECs with EMPs in the presence of palmitate for the last 3 h of EMP treatment; and *5*) HUVECs in the combined presence of EMPs and palmitate for 24 h.

Initially, HUVECs that were incubated with either 10^5^ or 10^6^ EMPs for 3 h exhibited a nonsignificant effect on NO production (Fig. 1*B* and Supplemental Fig. 1*B*), but sustained treatment for 24 h decreased A23187-stimulated NO production (*P* < 0.001; Fig. 1*C* and Supplemental Fig. 1*C*). As expected, palmitate-treated cells also exhibited significantly reduced A23187-stimulated NO production (*P* < 0.001; Fig. 1*B, C*); however, with the addition of palmitate during the last 3 h or throughout the 24 h of EMP treatment, EMPs had a restorative effect on A23187-stimulated NO production (*P* < 0.001; Fig. 1*C*).

### EMPs modulate mRNA and phosphorylation levels of eNOS and Akt under palmitate-induced oxidative stress

Previous studies have shown that eNOS acts *via* the PI3K pathway in endothelial cells (38, 39); therefore, to determine whether this pathway is modulated by EMP treatment, HUVECs were incubated with EMPs for 24 h and demonstrated no significant change in either eNOS and Akt mRNA expression (Supplemental Fig. 2) or protein phosphorylation (Fig. 1*D, E*). Palmitate treatment produced a marked decline in both Akt and eNOS mRNA and protein phosphorylation, which was rescued by EMP treatment under conditions of short-term (3 h) or long-term (24 h) oxidative stress (*P* < 0.001).

### EMPs protect against palmitate-induced ROS production and oxidative stress *in vitro*

**Figure 2.**
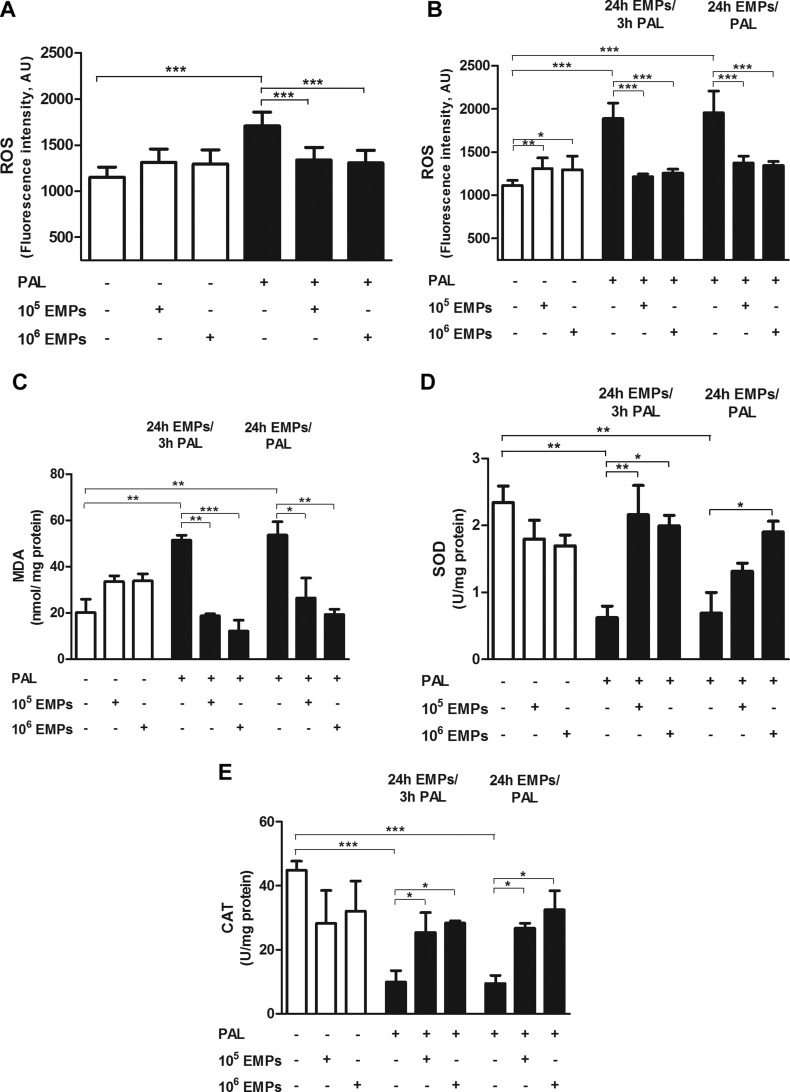
Effects of EMPs and/or palmitate (Pal) on ROS production and oxidative stress in HUVECs. *A*) EMPs produce no effect on ROS production from HUVECs after a 3-h treatment period, but exhibit a significant protective effect after Pal-induced oxidative stress. *B*) EMPs significantly increase ROS production after treatment for 24 h, but with the addition of Pal during the last 3 h (24 h EMPs/3 h Pal), or with EMPs and Pal for either 3 or 24 h (24 h EMPs/Pal), EMPs protect against Pal-induced ROS production. *C*–*E*) HUVECs that were treated with Pal for 3 and 24 h demonstrated a significant increase in MDA, a marker of lipid peroxidation (*C*), and concomitant decrease in the activity of SOD (*D*) and CAT (*E*). EMPs prevent Pal-induced lipid peroxidation and diminished the activity of antioxidant enzymes in HUVECs that were treated with either EMPs for 24 h with the addition of Pal during the last 3 h (24 h EMPs/3 h Pal) or with EMPs and Pal for 24 h (24 h EMPs/Pal). Results are means ± sem; *n* = 6–10 and analyzed using 1-way ANOVA. AU, arbitrary unit. *P < 0.05, **P < 0.01, ***P < 0.001.

We next investigated the effect of EMPs on ROS production, lipid peroxidation, and activity of antioxidant enzymes under homeostatic and palmitate-induced oxidative stress conditions. Although EMP treatment alone demonstrated no significant effect on ROS production in the short term (3 h; **Fig. 2*A***), a longer, 24-h incubation significantly increased ROS production (Fig. 2*B*; *P* < 0.05) but not to the levels that were achieved under palmitate-induced oxidative stress conditions. In contrast, EMPs significantly diminished ROS production under palmitate-induced oxidative stress (*P* < 0.001; Fig. 2*A, B*).

Of note, EMPs alone did not alter levels of the lipid peroxidation marker, MDA, compared with untreated cells (Fig. 2*C*). In support of ROS data, MDA levels were significantly increased (*P* < 0.01) under palmitate-induced oxidative stress, an effect that was significantly reduced after the administration of EMPs at both 10^5^ (*P* < 0.05) and 10^6^ (*P* < 0.01) doses.

EMP treatment alone demonstrated no difference in SOD and CAT activity compared with controls. Palmitate induced a significant decrease in the activity of both SOD (*P* < 0.01) and CAT (*P* < 0.001), which was rescued by EMP treatment (Fig. 2*D, E*).

### EMPs modulate NADPH oxidase activity in HUVECs in response to oxidative stress

**Figure 3.**
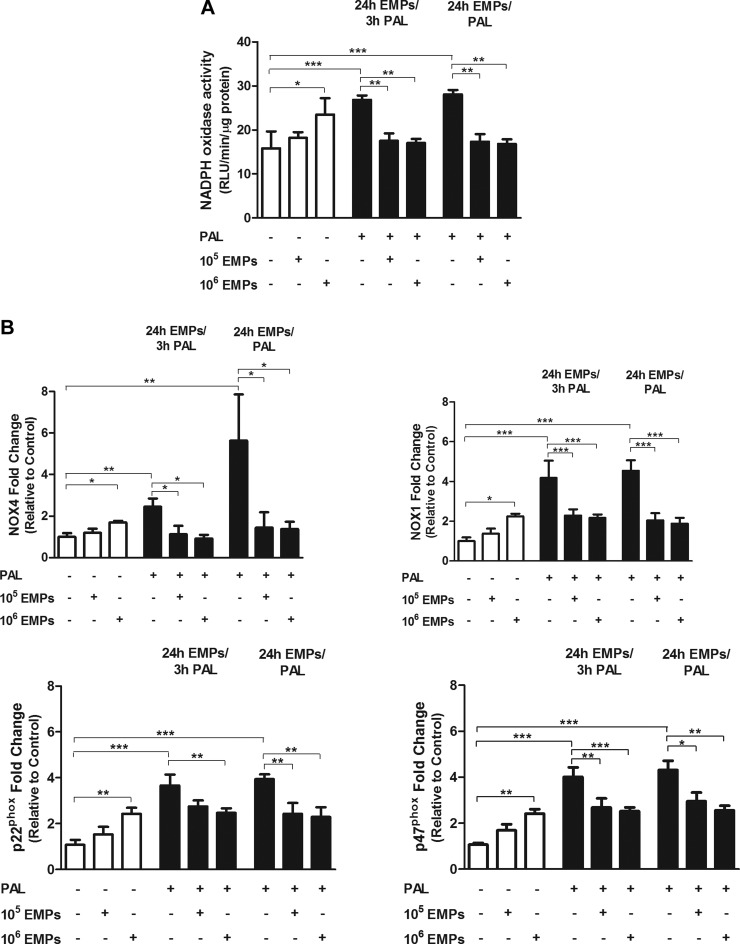
Effects of EMPs and/or palmitate (Pal) on NADPH oxidase in HUVECs. NADPH oxidase activity measured by lucigenin-enhanced chemiluminescence. Expression of NADPH oxidase subunits, NOX-4, NOX-1, p22^phox^, and p47^phox^, at the level of mRNA by RT-PCR. Palmitate and EMPs (10^6^) increase both NADPH oxidase activity (*A*) and expression of the enzyme subunits (*B*). EMPs reduce Pal-induced NADPH oxidase activation and expression in HUVECs that are treated with either EMPs for 24 h with the addition of Pal during the last 3 h (24 h EMPs/3 h Pal) or with EMPs and Pal for 24 h (24 h EMPs/Pal). Results are means ± sem; *n* = 6–8 and analyzed using 1-way ANOVA. RLU, relative luminescence units. *P < 0.05; **P < 0.01; ***P < 0.001.

To further interrogate the mechanism of EMP reduction of oxidative stress, the NADPH oxidase pathway was investigated. Although the 10^5^ EMP dose had no effect on NADPH oxidase activity over 24 h, the 10^6^ dose significantly increased enzyme activity compared with controls (*P* < 0.05; **Fig. 3*A***). Short- and long-term palmitate-induced oxidative stress conditions produced a significant increase in NADPH oxidase activity (*P* < 0.001), which was significantly decreased after EMP treatment at both doses (*P* < 0.01).

EMP treatment at the 10^5^ dose had no effect on mRNA abundance of the NADPH oxidase subunits, NOX4, NOX1, p47*^phox^* and p22*^phox^*, whereas the 10^6^ dose significantly increased mRNA expression of NOX4 (*P* < 0.05), NOX1 (*P* < 0.05), p47*^phox^* (*P* < 0.01), and p22*^phox^* (*P* < 0.01; Fig. 3*B*). Palmitate-induced oxidative stress conditions produced a significant up-regulation of NOX4 (*P* < 0.01), NOX1 (*P* < 0.001), p47*^phox^* (*P* < 0.001), and p22*^phox^* (*P* < 0.001), which were all significantly down-regulated after EMP treatment under the same conditions.

### Beneficial effects of EMPs against palmitate-induced oxidative stress *via* the Nrf2/ARE pathway *in vitro*

**Figure 4.**
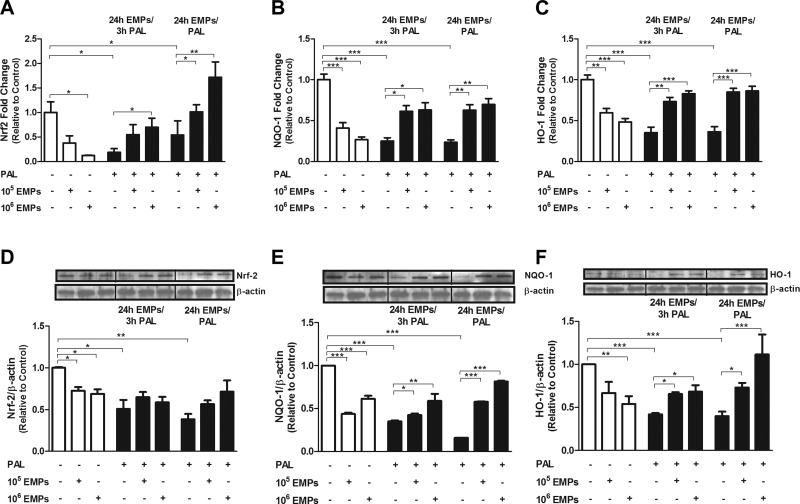
Effects of EMPs and/or palmitate (Pal) on Nrf2/ARE pathway in HUVECs. *A*–*F*) Effect of EMPs and/or Pal treatment on HUVECs with regard to Nrf2 (*A, D*), NQO-1 (*B*, *E*), and HO-1 (*C, F*) levels was determined by using quantitative RT-PCR and Western blotting. Treatment with either EMPs or Pal produces a significant decrease in both mRNA (*A–C*) and protein (*D–F*) expression of Nrf2, NQO1, and HO-1. EMPs increase expression of Nrf2, NQO1, and HO-1 in HUVECs that are treated with either EMPs for 24 h with the addition of Pal during the last 3 h (24 h EMPs/3 h Pal) or with EMPs and Pal for 24 h (24 h EMPs/Pal). Results are means ± sem; *n* = 6 and analyzed using 1-way ANOVA. *P < 0.05, **P < 0.01, ***P < 0.001.

To evaluate whether the downstream NRF2/ARE pathways are involved in the antioxidant effects of EMPs under oxidative stress conditions, mRNA and protein expression of Nrf2, NQO1, and HO-1 was determined by using quantitative RT-PCR and Western blotting, respectively (**Fig. 4**). EMP treatment at the 10^5^ dose for 24 h showed no change in Nrf2 mRNA abundance; however, Nrf2 protein levels were significantly decreased (*P* < 0.05). The 10^6^ EMP dose significantly decreased both Nrf2 mRNA (**Fig. 4*A***) and protein (Fig. 4*D*) levels. Under short- and long-term palmitate-induced oxidative stress conditions, a significant reduction in Nrf2 mRNA and protein levels was observed compared with homeostatic controls; however, EMPs under oxidative stress conditions caused a significant attenuation of this effect, in which Nrf2 mRNA abundance was higher, whereas Nrf2 protein levels, although slightly increased, were not significantly different compared with palmitate-treated cells.

NQO1 exhibited a similar expression pattern. Both 10^5^ and 10^6^ EMP doses significantly decreased the expression of both NQO1 gene and protein (Fig. 4*B*, *E*; *P* < 0.001). Palmitate-induced oxidative stress conditions also significantly diminished the mRNA and protein expression levels of NQO1 (*P* < 0.001), an effect that was abolished in the presence of EMPs.

HO-1 expression also followed this pattern. mRNA was significantly decreased (*P* < 0.001) after 10^5^ EMP treatment, whereas its protein level was unaffected. Treatment with 10^6^ EMPs for 24 h significantly reduced the mRNA (*P* < 0.001) and protein (*P* < 0.01) expression of HO-1 (Fig. 4*C, F*). A significant decrease in both HO-1 gene and mRNA and protein expression was observed under oxidative stress conditions (*P* < 0.001) with a marked up-regulation in the presence of EMPs in a dose-dependent manner.

### EMPs restore endothelium-dependent vasodilatation and NADPH oxidase *ex vivo*

**Figure 5.**
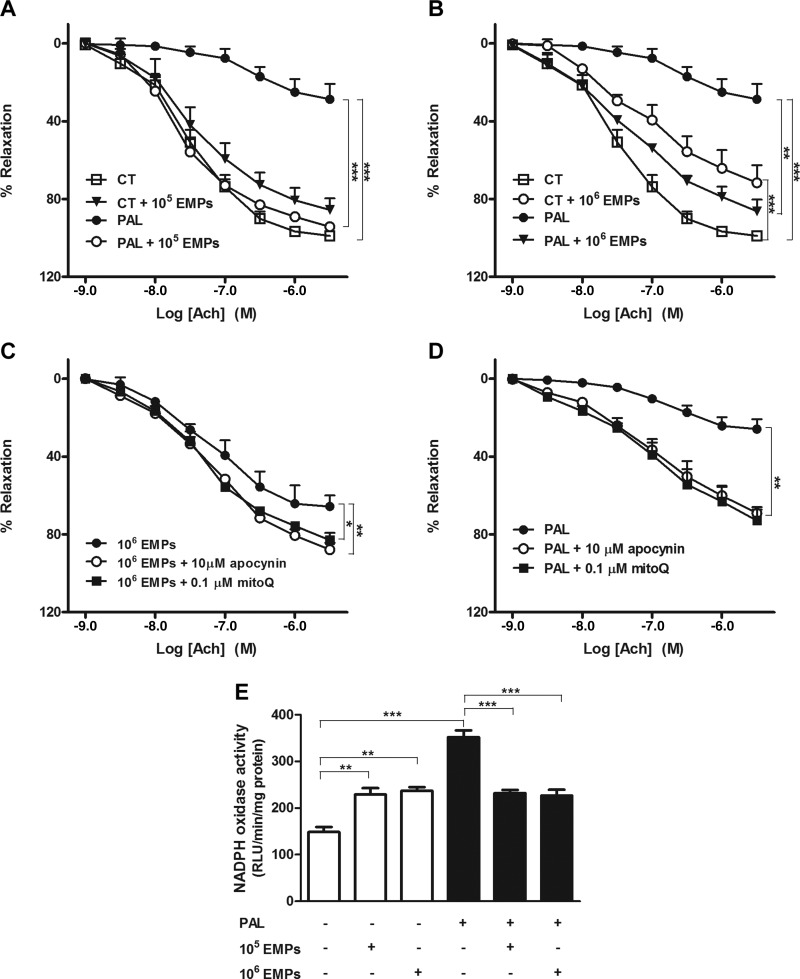
Effects of EMPs and/or palmitate (Pal) on endothelium-dependent vasodilatation and NADPH oxidase in mouse aortas. *A, B*) Treatment of aortic rings with either 10^6^ EMPs or Pal for 24 h diminished endothelium-dependent vasodilator responses to ACh. EMPs at both 10^5^ (*A*) and 10^6^ (*B*) doses improve the Pal-reduced endothelium-dependent vasodilatation. *C, D*) ROS are involved in endothelial dysfunction induced by EMPs (*C*) and Pal (*D*). MitoQ and apocynin improve the impaired aortic relaxation in response to ACh, induced by EMPs or Pal. *E*) NADPH oxidase activity increases in aortic rings that are treated with either EMPs or Pal for 24 h. In the presence of Pal, EMPs decrease NADPH oxidase activity. Results are means ± sem; *n* = 8–12. Data in panels *A*–*D* were analyzed using 2-way ANOVA and in panel *E* using 1-way ANOVA. CT, control; RLU, relative luminescence units. *P < 0.05; **P < 0.01; ***P < 0.001.

To validate *in vitro* studies in an *ex vivo* model, contractile analysis was performed in mouse aortic vessels. Aortas that were treated with 10^5^ EMPs demonstrated no significant difference in endothelium-dependent vasodilator responses to acetylcholine (ACh) compared with control aortas (**Fig. 5*A***); however, an increased dose of 10^6^ EMPs produced a significant decline in endothelium-dependent vasodilatation (Fig. 5*B*; *P* < 0.001). Palmitate-induced oxidative stress conditions over 24 h significantly diminished endothelium-dependent vasodilator responses to ACh, as expected (*P* < 0.001), and both doses of EMPs significantly attenuated this effect (*P* < 0.001). To validate the endothelial-dependent relaxation responses to ACh, the NOS inhibitor L-NAME was added, which abolished the relaxant response that was induced by ACh in all experimental groups as shown in Supplemental Fig. 3.

To examine whether ROS are involved in endothelial dysfunction induced by EMPs (Fig. 5*C*) and palmitate-induced oxidative stress conditions (Fig. 5*D*) in mouse aorta, the endothelium-dependent relaxant response to ACh in the presence of the mitochondrial antioxidant, mitoQ, or the NADPH oxidase inhibitor, apocynin, were analyzed. Both mitoQ and apocynin significantly improved the impaired aortic relaxation in response to ACh induced by EMPs or palmitate.

NADPH oxidase activity as measured by lucigenin-enhanced chemiluminescence (Fig. 5*E*) was also significantly increased by both EMPs and palmitate, an effect that was more pronounced in the presence of palmitate; however, under palmitate-induced oxidative stress conditions, EMPs attenuated the effect, producing a significant decrease in the activity of NADPH oxidase compared with oxidative stress conditions alone (*P* < 0.001).

## DISCUSSION

To our knowledge, this is the first report to demonstrate that EMPs restore the lipid-induced impairment of endothelium-dependent vasodilation and NO production. Moreover, these data point to the possible involvement of Akt/eNOS and Nrf2/ARE pathways in mediating the effects of EMPs on the endothelium. We also suggest when and how EMPs exert pathologic or protective effects on the endothelium.

Although Horn *et al.* (40) have suggested that circulating MPs retain functional eNOS by using immunoprecipitation, Western blot analysis, a Griess assay, and the fluorescent probe MNIP-Cu, we strengthen and expand these data by confirming more directly the presence of a functional eNOS by using the NO-sensitive fluorescent probe, DAF-2, and a specific pharmacologic inhibitor of NOS, L-NAME, that significantly prevented A23187-stimulated NO production from EMPs.

Although a short-term exposure of HUVECs to EMPs produced no change in A23187-stimulated NO production, a more sustained exposure of HUVECs with both doses of EMPs markedly diminished NO production. This suggests that other factors in the EMPs could mask the ability of EMP eNOS to function, which causes a decrease in eNOS activity and NO generation. Our data also add strength to the findings of Brodsky *et al.* (27), who first reported that EMPs decreased NO production in rat aortic rings, an effect that was attributed to increased O_2_^⋅−^ production.

In our palmitate-induced model of oxidative stress, we demonstrated diminished NO production *via* reduced Akt-dependent phosphorylation of eNOS at Ser^1177^, which supports the findings of previous reports (41, 42); however, the damaging effect of palmitate was attenuated when HUVECs were coincubated with EMPs. EMPs prevented the palmitate-induced decline in NO production *via* phosphorylation of both Akt and eNOS; therefore, EMPs exert paradoxical effects on eNOS activity and NO production in endothelial cells. While inducing endothelial cell damage to healthy cells, under an FFA insult, EMPs act to restore NO bioavailability.

It is well established that ROS scavenges NO and activates membrane oxidases, which increases the level of asymmetric dimethylarginine—an arginine decoy for active sites on eNOS and l-arginine transporters (43). In light of the fact that an increased production of O_2_^⋅−^ could play a role in the reduced bioavailability of NO (44), we investigated the effects of EMPs on ROS production. Our data demonstrate that under healthy conditions, EMPs had little effect on ROS production, levels of lipid peroxidation, or the activity of the antioxidant enzymes, SOD and CAT. We also demonstrated the validity of our oxidative stress model in that palmitate-enhanced ROS production, increased the lipid peroxidation product, MDA, and decreased the activity of the antioxidant enzymes, SOD and CAT. These effects were attenuated by EMPs. The potentiation of antioxidant defenses and normalization of ROS contributed to the restoration of endothelial function.

Another major player in ROS generation in the vasculature includes NADPH oxidases, and it has been reported that FFAs induce ROS production in the endothelium by increasing the activity of NADPH oxidases (8, 45). Here, we demonstrated that FFA increased the activity of NADPH oxidase, which correlated with an increased expression of its NOX1, NOX4, p22*^phox^*, and p47*^phox^* subunits. Of interest, EMPs protected against these effects and reduced NADPH oxidase activity, which correlated with reduced expression of NOX subunits.

To probe the downstream targets of ROS, we investigated the antioxidant transcription factor Nrf2/ARE pathway. Nrf2 is essential in cytoprotective mechanisms against oxidative stress and operates *via* transcriptional activation of ARE-dependent expression of antioxidant genes, including HO-1, NQO-1, SOD, and CAT (46, 47). We observed a significant decline in Nrf2 and its downstream genes and proteins when cells were treated with either EMPs or palmitate alone. Mann *et al.* (48) proposed that the basal activity of NADPH oxidase produces ROS, thereby activating Nrf2/ARE-mediated antioxidant gene expression to maintain redox homeostasis; however, we suggest that superfluous ROS production could lead to Nrf2 down-regulation in normal physiologic conditions. This hypothesis is based on the results of multiple studies that have demonstrated inhibition of the Nrf2 pathway under oxidative stress conditions. For example, Liu *et al.* (49) reported that oscillating high glucose enhanced oxidative stress and apoptosis in human coronary artery endothelial cells as a result of the inhibition of the Nrf2-HO-1 pathway. Mahmoud and Al Dera (50) demonstrated that cyclophosphamide-induced oxidative stress significantly abolished the activation of the liver Nrf2 pathway. Finally, the Mann laboratory has also shown that disturbed shear stress on the vascular wall, which results from oscillatory blood flow, diminishes the Nrf2-mediated activation of ARE-linked genes (51). Moreover, we have recently reported that palmitate down-regulated the Nrf2/ARE/HO-1 signaling pathway in HUVECs (37).

In contrast, under palmitate insult, we observed that EMPs significantly activated Nrf2 and its downstream proteins, HO-1 and NQO-1. This could explain, in part, the protective mechanism of EMPs against lipid-induced endothelial dysfunction, which supports the findings of other studies (52, 53) where, for example, HO-1 is known to degrade the pro-oxidant heme to biliverdin, which is subsequently converted to the radical scavenger, bilirubin (54). Bilirubin, in turn, may directly inhibit NADPH oxidase by interrupting the assembly and activation of the enzyme in endothelial cells both *in vitro* and *in vivo* (55, 56). Furthermore, interplay between Nrf2 and eNOS is not fully understood. Activation of Nrf2 by CDDO-IM (2-cyano-3,12-dioxooleana-1,9-dien-28-oic imidazolide) increased the amount of bioavailable NO in HUVECs (57). Activation of Nrf2 enhanced NO generation in human glomerular endothelial cells, as recently reported by Luo *et al.* (58). Mann *et al.* (48) and Fledderus *et al.* (59) hypothesized that augmented eNOS activity enhances ARE-linked gene transcription.

Having demonstrated a clear protective mechanism for EMPs under oxidative stress *in vitro*, the same experimental design was carried out *ex vivo* in mouse aortic rings. Previous studies have demonstrated impaired ACh-induced relaxation in mouse aorta that was treated with T lymphocyte-derived MPs *via* activation of xanthine oxidase-induced ROS (60). In addition, Boulanger *et al.* (61) reported diminished endothelial NO-mediated relaxation in normal rat aortic rings that were treated with circulating MPs from patients with myocardial infarction. Here, we showed that the inhibition of NADPH oxidase and mitochondrial ROS production by the pharmacologic agents, apocynin and mitoQ, respectively, reduced the ability of EMPs to diminish ACh-induced relaxation. These results suggest that EMPs exert their effects on ROS production and vascular relaxation, mainly *via* ROS produced by NADPH oxidase and mitochondria. Our findings were further validated *ex vivo* by the increased activity of NADPH oxidase in EMP-treated mouse aortic rings.

Under palmitate-induced oxidative stress, mouse aortic rings exhibited impaired ACh-induced relaxation. Previous research from the Duarte laboratory has demonstrated that palmitate-induced endothelial dysfunction is associated with increased ROS production (41). Use of the NADPH oxidase inhibitor, apocynin, and the mitochondrial antioxidant, mitoQ, partially counteracted the palmitate-induced impairment of endothelium-dependent relaxation, which strengthened their findings (41); however, in accordance with our *in vitro* studies, EMPs effectively improved endothelium-dependent relaxation and decreased the activity of NADPH oxidase in palmitate-induced mouse aortas. In light of these data, it seems likely that EMPs improved vascular reactivity *via* inhibition of ROS production and an increased NO bioavailability. Incubation of aortic rings from all experimental groups with a high concentration of the NOS inhibitor, L-NAME, almost abolished ACh-induced relaxation, which indicated that it is completely dependent on eNOS-derived NO.

Our findings raise a number of key questions that future work will help to explore. Foremost is identification of the bioactive mediators that are contained within EMPs. Screening EMPs for microRNAs and proteins may clarify the different effects observed in our experiments. EMPs are produced under homeostatic conditions, albeit not in the quantities used in our experiments (61), and different conditions may alter their behavior. Classically, EMPs have been considered to be pathogenic mediators, but we suggest that, in fact, they have a dual role that is aimed at maintaining endothelial health under conditions of stress, while also potentially being pathogenic, depending on their environment. In support of this, whereas EMPs are elevated in cardiovascular disease (26), they have been shown to be protective in sepsis (62), which suggests that their number and role alters depending on the pathologic insult. In addition, how EMPs interact with target cells may reveal them to be therapeutic targets to modify disease pathogenesis. MPs are reported to be taken up by membrane fusion, receptor-mediated cell signaling, or phagocytosis (23, 24). We have followed the protocol described in the position paper by Lacroix *et al.* (63) to isolate EMPs from HUVECs, but it is likely that EMPs from different vascular beds may have different, if not parallel, effects that will be investigated in future studies. Indeed, all *in vitro* models are limited in what we can conclude about EMP pathophysiology, as defined by other groups (64, 65).

**Figure 6.**
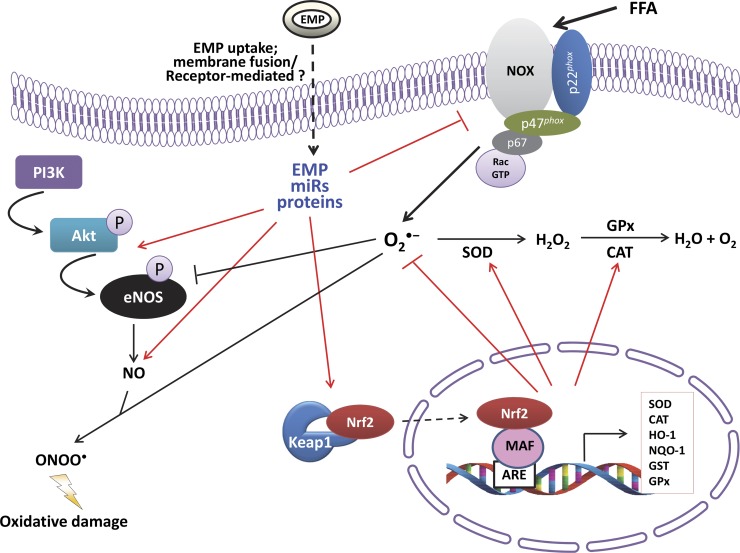
Schematic diagram demonstrating the molecular mechanisms by which EMPs inhibit FFA-induced endothelial dysfunction. O_2_^⋅−^, superoxide radical; ONOO^⋅^, peroxynitrite; GPx, glutathione peroxidase; GST, glutathione-*S*-transferase.

Nonetheless, our results describe a novel role for EMPs. Our mechanistic evidence suggests that this vascular benefit is likely to result from an enhancement of Nrf2 expression, which leads to the up-regulation of enzymatic antioxidants, attenuating ROS production and lipid peroxidation under high FFA conditions. Furthermore, to our knowledge, we provide the first evidence of functional eNOS in EMPs and its ability to generate NO. Overall, we conclude that EMPs are vectors of paradoxical information in the endothelium with a role in both health and disease (**Fig. 6**); thus, EMP function is dependent on the pathologic context and mechanisms and sites of formation. Future work must focus on the analysis of EMP content, which may hold unique prognostic information to stratify patients for appropriate therapy on the basis of their risk of atherothrombotic disease.

## Supplementary Material

Supplemental Data

## Abbreviations

ACh: acetylcholine
ARE: antioxidant response element
BSA: bovine serum albumin
CAT: catalase
DAF-2: diaminofluorescein-2
EMP: endothelial microparticle
FFA: free fatty acid
HO-1: heme oxygenase-1
L-NAME: *N*-nitroarginine methyl ester
MDA: malondialdehyde
MP: microparticle
Nrf2: NF-erythroid 2–related factor 2
NQO1: NAD(P)H:quinone oxido-reductase 1
ROS: reactive oxygen species
SOD: superoxide dismutase
