# Supplementary material for: Endothelial microparticles prevent lipid-induced endothelial damage via Akt/eNOS signaling and reduced oxidative stress
Source: FASEB J. 2017 Jul 7;31(10):4636–48. doi: 10.1096/fj.201601244RR (PMC5714503; doi:10.1096/fj.201601244RR)
Supplement: Supplemental Data [file supp_31_10_4636__index.html]

Endothelial microparticles prevent lipid-induced endothelial damage via Akt/eNOS signaling and reduced oxidative stress — Supplemental Data 

# Endothelial microparticles prevent lipid-induced endothelial damage *via* Akt/eNOS signaling and reduced oxidative stress

## Supplemental Data

- Supplemental Data
- Supplemental Data
- Supplemental Data
- Supplemental Data
