# Supplementary material for: Endothelial microparticles prevent lipid-induced endothelial damage via Akt/eNOS signaling and reduced oxidative stress
Source: FASEB J. 2017 Jul 7;31(10):4636–48. doi: 10.1096/fj.201601244RR (PMC5714503; doi:10.1096/fj.201601244RR)
Supplement: Supplemental Data [file supp_fj.201601244RR_Supplemental_Data1.docx]

**SUPPLEMENTAL MATERIAL AND METHODS A; Endothelial microparticles prevent lipid-induced endothelial damage via Akt/eNOS signaling and reduced oxidative stress**

**EMP quantification**

Briefly, samples were diluted in Annexin V binding buffer and Flow-Count™ Fluorospheres (Beckman Coulter, High Wycombe, UK) were added. Samples were labeled with AnnexinV-FITC (Becton Dickinson) and incubated on ice in the dark for 15 min. Flow cytometry was set up and MP gates were defined using sizing beads of 0.16uM, 0.2uM, 0.24uM and 0.5uM (Megamix-Plus SSC, Stago, Marseille). Threshold was set on SSC as recommended by the manufacturer of the sizing beads for maximum accuracy in Becton Dickinson instruments. These beads accurately allow the detection of MPs in the size range of 0.1uM – 1uM. Sizing beads are fluorescent in the FITC channel and therefore, they were only used to identify the correct voltages and gating strategy. Flow cytometry analysis was performed by gating the counting fluorospheres on Forward scatter (FSC) vs Side Scatter (SSC) (linear scale). Samples were acquired at low flow rate until 1000 counting beads were detected. AnnexinV-FITC positive events were selected as MPs.

**Measurement of lipid peroxidation**

Following incubation of HUVECS with palmitate and/or EMPs, the cells were washed and homogenized. SDS lysis solution was added to the HUVECS samples or MDA standards. Samples and standards were then incubated with thiobarbituric acid for 45 minutes at 95°C. Samples were brought to room temperature and centrifuged at 1000 x g for 15 min. Supernatants were removed to a 96-well plate and absorbance was measured spectrophotometrically at 532 nm using a microplate reader (BioTek), and the results were expressed as nmol MDA/mg protein.

**Real-time PCR**

Real-time PCR reaction was carried out using the following conditions; 10 min at 95°C, followed by 40 cycles of 30 sec at 95ºC, 60 sec at annealing temperature of respective primer set, and extension for 30 sec at 72ºC. To evaluate the specificity and quality of PCR amplifications, melting curve analysis and an agarose gel electrophoresis-based quality check were performed. The cycle threshold (CT) values were analyzed using the 2^-ΔΔCt^ method.

**Western blot analysis**

The protein concentration was determined using a Bicinchoninic acid (BCA) protein assay kit (Pierce Biotechnology). Equal amounts of protein (30 μg) were denatured and separated by sodium dodecyl sulphate (SDS)-polyacrilamide gel electrophoresis. Proteins were transferred to polyvinylidene difluoride (PVDF) membranes, blocked for 1h in Tris-Buffered Saline Tween-20 (TBST) with 5% non-fat milk, and probed with primary rabbit polyclonal anti- phospho-eNOS (Cell Signaling), rabbit anti-eNOS, rabbit anti-phospho-protein kinase B (Akt), rabbit anti-Akt (Santa Cruz Biotechnology), rabbit anti-HO-1 (Stressgen), rabbit anti-Nrf2, rabbit anti-NQO-1 (Santa Cruz Biotechnology) and mouse anti-β-actin (Sigma), with gentle agitation overnight at 4ºC. The membranes were washed with TBST and incubated with the correspondent secondary peroxidase conjugated antibodies for 1h at room temperature. The membranes were then visualized using ECL (Amersham Pharmacia Biotech, Amersham, UK) and densitometric analysis was performed using ImageJ (version 1.32j, NIH, <http://rsb.info.nih/ij/>).
