# Supplementary material for: Endothelial microparticles prevent lipid-induced endothelial damage via Akt/eNOS signaling and reduced oxidative stress
Source: FASEB J. 2017 Jul 7;31(10):4636–48. doi: 10.1096/fj.201601244RR (PMC5714503; doi:10.1096/fj.201601244RR)
Supplement: Supplemental Data [file supp_fj.201601244RR_Supplemental_Data2.docx]

**SUPPLEMENTAL MATERIAL AND METHODS B; Endothelial microparticles prevent lipid-induced endothelial damage via Akt/eNOS signaling and reduced oxidative stress**

**Ex vivo assessment of mouse aortic ring vascular reactivity**

Mice were culled using cervical dislocation and aortas were dissected in sterile PBS, cleared of periadventitial tissue and cut transversely into 2.0-mm rings. Mouse thoracic aortic (MTA) rings were incubated in Dulbecco’s modified Eagle’s medium (DMEM) supplemented with 10% FBS, plus 100 U/mL penicillin, and 100 μg/mL streptomycin. MTA rings were then incubated for 24 h in serum-free DMEM containing 2% fatty acid-free BSA (control) or palmitate conjugated BSA (100μM) in either the presence or absence of 10^5^ or 10^6^ EMPs. Vascular rings were handled carefully to avoid damage to the inner surface, transferred to a chamber filled with fresh Kreb’s solution and mounted in a myograph (model 610M, Danish Myo Technology, Aarhus, Denmark) for isometric tension measurement, as previously described (36). Concentration-relaxation response curves to acetylcholine (ACh; 10^-9^ M -10^-5^ M) were performed in aortic rings pre-contracted by U46619 (10^-8^ M) in control or L-NAME (100μM)-treated aortic rings. To examine whether ROS contribute to endothelial dysfunction induced by palmitate and EMPs in mouse aorta, responses to ACh were studied after incubation with the mitochondrial antioxidant mitoQ (0.1µM) or the NADPH oxidase inhibitor apocynin (10µM), and incubated for 60 min before the addition of U46619. Relaxant responses to ACh were expressed as a percentage of pre-contraction. 8-12 MTA rings, each from a different mouse were used per group.

**Supplementary Table I. Primer sequences used for qRT-PCR.**

| **Gene** | **Sequence (5'-3')** | **Accession number** | **Tm** |
| --- | --- | --- | --- |
| NOS3 | F: GCATCACCTATGACACCCTC  R: CATGTACCAGCCACTGAAGG | [XM_006716002.2](http://www.ncbi.nlm.nih.gov/entrez/viewer.fcgi?db=nucleotide&id=767947907) | 62.6  63.1 |
| AKT | F: AATGGACAGAAGCTATCCAGGC  R: TGATGGGTTGTAGAGGCATCC | [NM_005465](http://www.ncbi.nlm.nih.gov/entrez/query.fcgi?cmd=Search&db=Nucleotide&term=NM_005465) | 61.8  61.3 |
| NOX4 | F: CAGATGTTGGGGCTAGGATTG  R: GAGTGTTCGGCACATGGGTA | [NM_001143836](http://www.ncbi.nlm.nih.gov/entrez/query.fcgi?cmd=Search&db=Nucleotide&term=NM_001143836) | 60.2  61.9 |
| NOX1 | F: CTGTTGCCTAGAAGGGCTCC  R: CGGCTGCAAAACCCAAGGAT | [NM_001271815.1](http://www.ncbi.nlm.nih.gov/entrez/viewer.fcgi?db=nucleotide&id=425854819) | 60.11  61.54 |
| p22*^phox^* | F: GATCGAGTGGGCCATGT  R: TGCTTGATGGTGCCTCC | [NM_000101.3](http://www.ncbi.nlm.nih.gov/entrez/viewer.fcgi?db=nucleotide&id=371941004) | 56.16  56.73 |
| p47*^phox^* | F: AAGTGGTTTGACGGGCAG  R: TGGACGGAAAGTAGCCTG | [XM_005250543.3](http://www.ncbi.nlm.nih.gov/entrez/viewer.fcgi?db=nucleotide&id=767948532) | 57.84  56.24 |
| NRF2 | F: GGTTCCAAGTCCAGAAGCCA  R: GGTTGGGGTCTTCTGTGGAG | [NM_001145413.2](http://www.ncbi.nlm.nih.gov/entrez/viewer.fcgi?db=nucleotide&id=372620348) | 59.89  59.96 |
| NQO-1 | F: TGAAAGGCTGGTTTGAGCGA  R: TCCAGGCGTTTCTTCCATCC | [NM_001025433.1](http://www.ncbi.nlm.nih.gov/entrez/viewer.fcgi?db=nucleotide&id=70995395) | 60.18  60.04 |
| HO-1 | F: AGTCTTCGCCCCTGTCTACT  R: CTTCACATAGCGCTGCATGG | [NM_002133.2](http://www.ncbi.nlm.nih.gov/entrez/viewer.fcgi?db=nucleotide&id=298676487) | 59.96  59.69 |
| GAPDH | F: GGAGCGAGATCCCTCCAAAAT  R: GGCTGTTGTCATACTTCTCATGG | NM_001256799 | 61.6  60.9 |
