# Supplementary material for: Endothelial microparticles prevent lipid-induced endothelial damage via Akt/eNOS signaling and reduced oxidative stress
Source: FASEB J. 2017 Jul 7;31(10):4636–48. doi: 10.1096/fj.201601244RR (PMC5714503; doi:10.1096/fj.201601244RR)
Supplement: Supplemental Data [file supp_fj.201601244RR_Supplemental_Data3.docx]

**
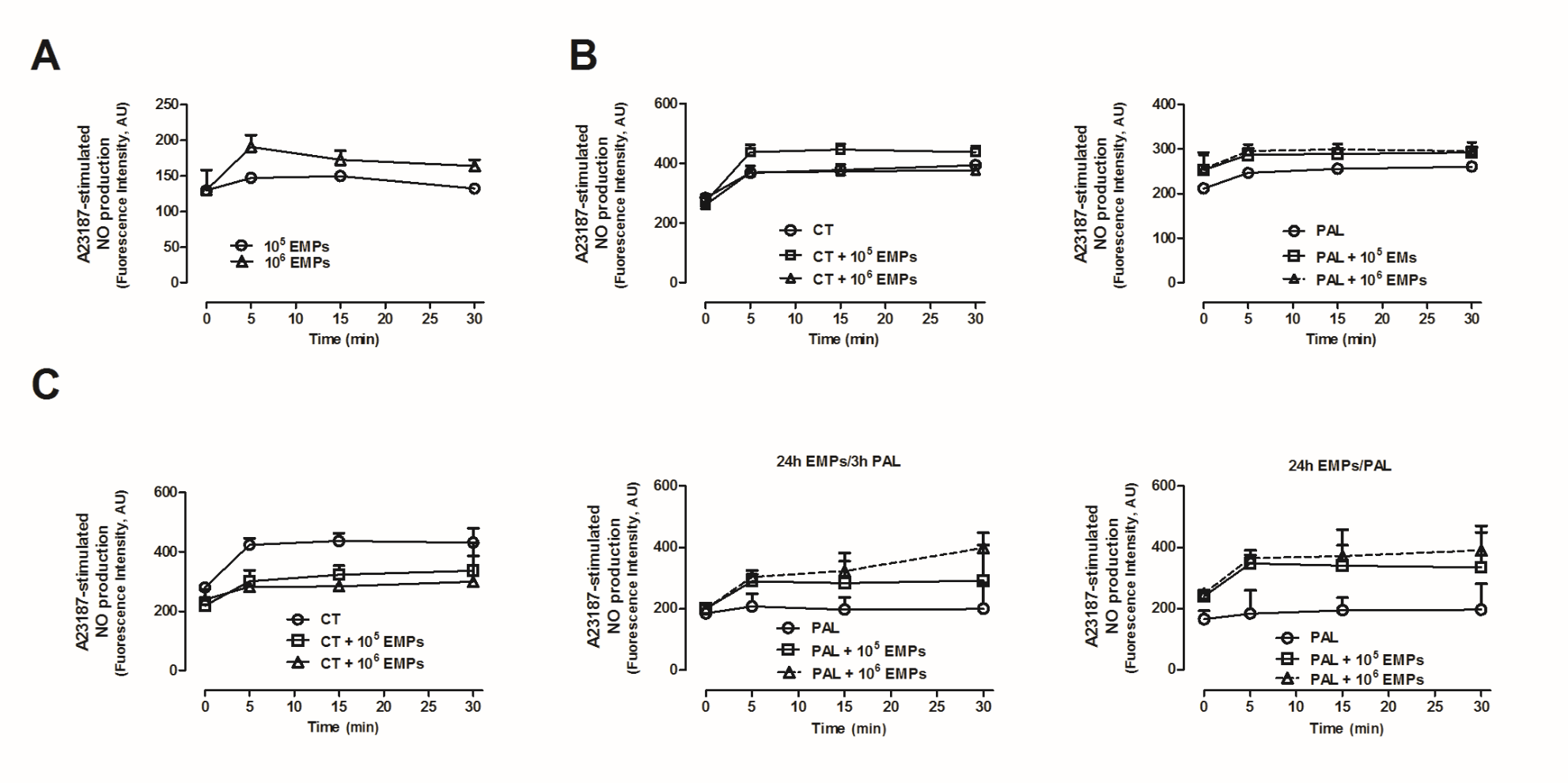
**

**Supplementary Figure SI.** **EMPs express a functional eNOS and elevate NO in palmitate-induced HUVECs.** (**A**) EMPs were incubated with L-arginine for 5 min at 37ºC followed by addition of DAF-2 and NO production was determined. EMPs produce eNOS-derived NO in a concentration dependent manner. (**B**) HUVECs incubated with EMPs for 3h showed no changes in NO production, whereas EMPs prevented palmitate-induced decline in NO production. (**C**) Palmitate and EMPs diminish NO production. Treatment with either EMPs for 24h with the addition of palmitate during the last 3h (24h EMPs/3h PAL) or EMPs and palmitate for 24h (24h EMPs/PAL) protects against palmitate-induced reduction in NO production. Results are mean ± SEM; N = 8-12. EMPs, endothelial microparticles; CT, control; PAL, palmitate; NO, nitric oxide.
