# Supplementary material for: Endothelial microparticles prevent lipid-induced endothelial damage via Akt/eNOS signaling and reduced oxidative stress
Source: FASEB J. 2017 Jul 7;31(10):4636–48. doi: 10.1096/fj.201601244RR (PMC5714503; doi:10.1096/fj.201601244RR)
Supplement: Supplemental Data [file supp_fj.201601244RR_Supplemental_Data4.docx]

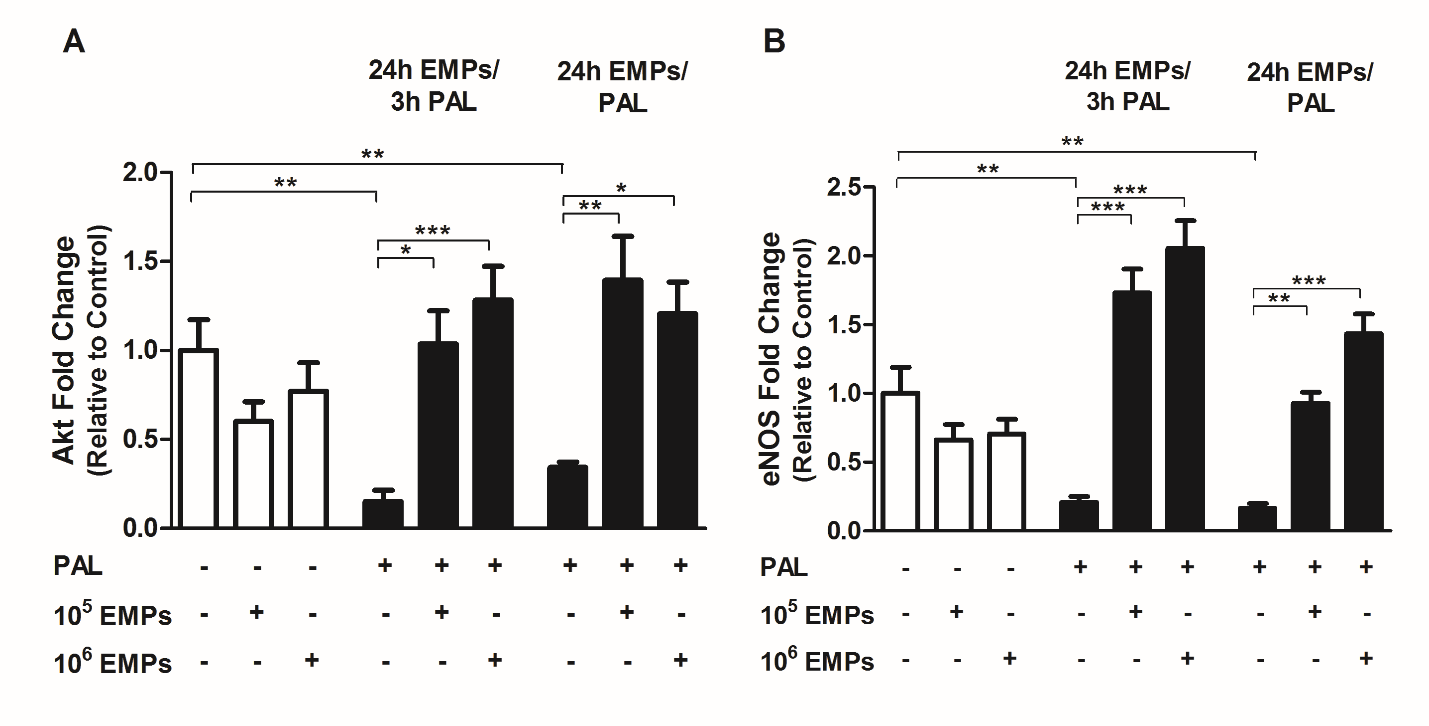


**Supplementary Figure SII. Effects of EMPs and/or palmitate on mRNA expression of Akt and eNOS.** Treatment of HUVECs with 100µM palmitate for either 3 or 24h decreases mRNA expression levels of (**A**) Akt and (**B**) eNOS. EMPs up-regulate Akt and eNOS expression in HUVECs treated with either EMPs for 24h with the addition of palmitate during the last 3h (24h EMPs/3h PAL) or EMPs and palmitate for 24h (24h EMPs/PAL). Results are mean ± SEM; N = 6 and analysed using one-way ANOVA. *P<0.05, **P<0.01 and ***P<0.001. EMPs, endothelial microparticles; PAL, palmitate; Akt, protein kinase B; eNOS, endothelial nitric oxide synthase.


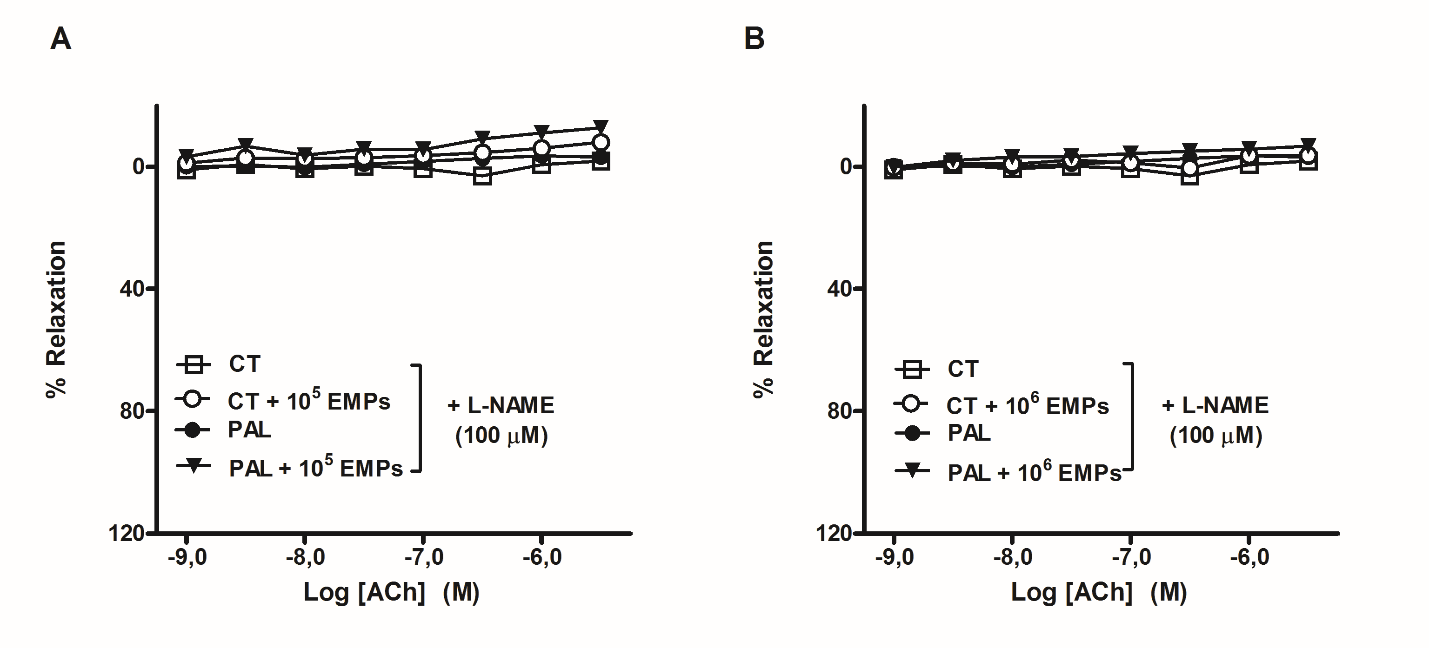


**Supplementary Figure SIII.** **L-NAME inhibits the** **endothelium-dependent vasodilatation in mice aortas treated with EMPs and/or palmitate.** Results are mean ± SEM; N = 8-12 and analysed using two-way ANOVA *P<0.05, **P<0.01 and ***P<0.001. EMPs, endothelial microparticles; CT, control; PAL, palmitate; ACh, acetylcholine.
